# Muscle oxygenation in Type 1 diabetic and non-diabetic patients with and without chronic compartment syndrome

**DOI:** 10.1371/journal.pone.0186790

**Published:** 2017-10-23

**Authors:** Patrik Gustafsson, Albert G. Crenshaw, David Edmundsson, Göran Toolanen, Sead Crnalic

**Affiliations:** 1 Department of Surgical and Perioperative Sciences (Orthopaedics), Umeå University, Umeå, Sweden; 2 Centre for Musculoskeletal Research, Department of Occupational and Public Health Sciences, Faculty of Health and Occupational Studies, University of Gävle, Gävle, Sweden; Tokai University, JAPAN

## Abstract

**Background:**

Type 1 diabetic patients and non-diabetic patients were referred for evaluation for chronic exertional compartment syndrome (CECS) based on clinical examination and complaints of activity-related leg pain in the region of the tibialis anterior muscle. Previous studies using near-infrared spectroscopy (NIRS) showed greater deoxygenation during exercise for CECS patients versus healthy controls; however, this comparison has not been done for diabetic CECS patients.

**Methods:**

We used NIRS to test for differences in oxygenation kinetics for Type 1 diabetic patients diagnosed with (CECS-diabetics, n = 9) versus diabetic patients without (CON-diabetics, n = 10) leg anterior chronic exertional compartment syndrome. Comparisons were also made between non-diabetic CECS patients (n = 11) and healthy controls (CON, n = 10). The experimental protocol consisted of thigh arterial cuff occlusion (AO, 1-minute duration), and treadmill running to reproduce symptoms. NIRS variables generated were resting StO_2_%, and oxygen recovery following AO. Also, during and following treadmill running the magnitude of deoxygenation and oxygen recovery, respectively, were determined.

**Results:**

There was no difference in resting StO_2_% between CECS-diabetics (78.2±12.6%) vs. CON-diabetics (69.1±20.8%), or between CECS (69.3±16.2) vs. CON (75.9±11.2%). However, oxygen recovery following AO was significantly slower for CECS (1.8±0.8%/sec) vs. CON (3.8±1.7%/sec) (P = 0.002); these data were not different between the diabetic groups. StO_2_% during exercise was lower (greater deoxygenation) for CECS-diabetics (6.3±8.6%) vs. CON-diabetics (40.4±22.0%), and for CECS (11.3±16.8%) vs. CON (34.1±21.2%) (P<0.05 for both). The rate of oxygen recovery post exercise was faster for CECS-diabetics (3.5±2.6%/sec) vs. CON-diabetics (1.4±0.8%/sec) (P = 0.04), and there was a tendency of difference for CECS (3.1±1.4%/sec) vs. CON (1.9±1.3%/sec) (P = 0.05).

**Conclusion:**

The greater deoxygenation during treadmill running for the CECS-diabetics group (vs. CON-diabetics) is in line with previous studies (and with the present study) that compared non-diabetic CECS patients with healthy controls. Our findings could suggest that NIRS may be useful as a diagnostic tool for assessing Type 1 diabetic patients suspected of CECS.

## Introduction

Chronic exertional compartment syndrome (CECS) primarily occurs in subjects that may or may not be athletes [1] that engage in running and walking exercise. The primary symptom is effort-related leg pain in an isolated muscle compartment that is recurrent, and the diagnosis is often verified by assessing intramuscular pressure (IMP) before and after exercise. In general, IMP for CECS subjects remains elevated for a prolonged time after exercise [2]. The aetiology of CECS is unclear but muscle ischemia secondary to elevated IMP is the most adopted theory.

Direct measurement of IMP involves an invasive technique that entails insertion of a needle or catheter into the muscle compartment of interest. In attempts to improve patient comfort and aid in diagnosis, clinicians and researchers have examined non-invasive techniques. For example, Mohler et al. [3] compared IMP measurements with near infrared spectroscopy (NIRS) in the anterior compartment of CECS patients versus healthy controls.

NIRS is a non-invasive technique whose signals represent the dynamic balance between oxygen delivery and oxygen consumption [4]. It is based on the principle of differential absorption properties of oxygenated and deoxygenated haemoglobin in the near-infrared range of the light spectrum. Some commonly reported variables in NIRS evaluations are changes in oxygenation, and re-oxygenation rate or recovery as during and following muscle activity, respectively. Further, when applying cuff occlusion, information on blood flow, oxygen consumption and oxygen delivery can be obtained [5,6].

In a recently published review three papers dealing with NIRS and CECS were identified [7]. Taken together the studies showed that CECS patients, in comparison to healthy controls, demonstrated greater relative deoxygenation during exercise and in one of the three studies CECS patients showed a longer time to re-oxygenation post-exercise [3]. Thus, an essential conclusion reached in the aforementioned review paper, albeit for a limited number of studies, was that there was sufficient evidence to propose that NIRS is a useful non-invasive method for assessing CECS [7].

A forthcoming group of interest for CECS evaluation is diabetic patients that experience debilitating leg pain with exertion. In a previous study, Edmundsson and Toolanen [8] confirmed CECS in a number of diabetic patients that had attended the clinic with lower leg pain presumed due to claudication. In another study from the same research group, the authors reported that CECS diabetic patients had higher IMP and endured a shorter walking distance before incapacitating leg pain than for non-diabetic patients with CECS [9]. Poor circulation in the feet and legs are hallmark signs of diabetic patients. More specifically these patients are prone to vascular disease [10] and intermittent claudication [11]. For non-diabetic CECS patients the greater deoxygenation during exercise, in comparison to healthy controls, is attributed to the elevated IMP. How oxygenation kinetics is impacted for diabetic patients with CECS has not been investigated.

In the present study we applied a protocol that entailed cuff occlusion and treadmill running to test the extent of differences in oxygenation kinetics between Type 1 diabetic patients with CECS compared to those without CECS. An inclusive question we had was whether NIRS could be used as a tool to distinguish between Type 1 diabetic patients with CECS and diabetic patients without CECS. To put our objective in perspective we also included a group of non-diabetic CECS patients and compared these with healthy controls; these data also served to elucidate on previous studies of this type. We hypothesized that deoxygenation during exercise would be greater for both CECS groups compared to their respective counterparts.

## Methods

### Subjects

During a 3-year period consecutive Type 1 diabetic patients and non-diabetic patients at Umea University Hospital in Northern Sweden were referred for evaluation for CECS based on clinical examination and complaints of activity-related leg pain in the region of the tibialis anterior muscle. The patients were non-elite athletes that on occasion attended the gym for training and/or participated in recreational running. A subsequent clinical assessment was done that included measurement of intramuscular pressure (IMP) and a treadmill exercise test to reproduce symptoms. For experimental purposes a probe for monitoring oxygenation was placed on the painful muscle region. IMP and oxygenation procedures are described below. Initially there were 15 diabetic subjects and 18 non-diabetic subjects tested and therefore these subjects performed all aspects of the experimental protocol. Of these, 10 diabetic subjects (CECS-diabetics) and 11 non-diabetic subjects (CECS) tested positive for CECS and were thus used in the study. In addition to the onset of pain symptoms that occurred during the treadmill exercise test for our positive CECS subjects, elevated IMP was a definitive factor in the diagnosis of CECS as follows: IMP pre exercise > 15 mm Hg, and IMP after treadmill running at 1 minute post exercise > 30 mm Hg, and at 5 minutes post exercise > 20 mm Hg. These IMP criteria are as suggested by Pedowitz et al. [2] and are commonly used in our clinic. Two additional groups that were non-CECS were recruited as respective controls that consisted of subjects that were Type 1 diabetics (designated as control diabetes, CON-diabetics) and healthy non-diabetic subjects (controls, CON). All of the diabetic patients (CECS-diabetics and CON-diabetics) were taking daily doses of insulin. Table 1 shows anthropometric and blood pressure data for all subjects. All participants were given verbal information, and provided their verbal consent to participate prior to the study. The study was approved by the regional ethical board in Umea, Sweden (Dnr 06-056M) in accordance to the declaration of Helsinki on the use of human subjects in research. The study was conducted at the Laboratory of Clinical Physiology at the University Hospital in Umea, Sweden.

**Table 1:** Anthropometric data.

	CECS-D (n = 9) females = 5	CON-D (n = 10) females = 5	CECS (n = 11) females = 6	CON (n = 10) females = 5
Age (yrs)	39 (17)	40 (11)	28 (10)	31 (7)
Height (cm)	178 (6)	174 (7)	176 (10)	178 (9)
Weight (kg)	80 (8)	74 (7)	73 (8)	73 (14)
Blood pressure-Systolic (mmHg)	135 (15)	123 (13)	116 (8)	111 (6)
Blood pressure-Diastolic (mmHg)	78 (6)	71 (11)	65 (8)	65 (5)

Data represent mean values (SD). CECS-D–CECS-diabetics; CON-D–CON-diabetics.

### Experimental procedures

Anthropometric and blood pressures measurements (conventional means with upper arm cuff) were done when subject first entered the laboratory, and before the start of the experimental procedures. The experimental procedures were conducted in the order of the following: (1) Intramuscular pressure (IMP) catheters and near infrared spectroscopy (NIRS) probes were placed, and data were collected at rest with the subject lying still in a supine position; (2) a pneumatic cuff was placed around the thigh and inflated to 250 mmHg (arterial occlusion) and maintained for 1 minute to record the resting oxygen consumption rate; (3) the cuff pressure was abruptly released and the subjects rested quietly for 30 seconds to record oxygen recovery, after which the cuff was removed; (4) the subject stood up and performed exercise on a treadmill that began at a slow pace (~ 8 km/h) and after 4 min was increased in speed (~ 10 km/h) and incline (elevated 4°) in an attempt to reproduce leg pain; the duration of running was 9.5 minutes (median) for CECS and 10.5 minutes for CECS-diabetic patients, and was 12 minutes for all control subjects; oxygenation responses were recorded throughout the exercise; (5) subjects returned to a supine position for post-exercise recording of IMP and oxygenation data (up to 5 minutes).

### IMP technique

IMP measurements were made for the CECS-diabetics and CECS groups only. IMP measurements were not done for the controls due to the invasive nature of the procedure and because IMP was used to clinically assess the presence of compartment syndrome and was not an integral part of the experimental questions we investigated. Catheters were inserted aseptically (without local anaesthesia) into the tibialis anterior (TA) muscle with the subject in a supine position. First a Teflon cannula was inserted into the TA in a distal direction at a site that was 2 cm lateral to the bottom of the tibial tuberosity. The insertion angle was 30 degrees to the skin. Then the IMP catheter (1.05 mm in diameter, Myopress; Athos Medical, Höör, Sweden) was threaded through the cannula and placed at a depth of 15–20 mm beneath the fascia. The catheter was connected via a Teflon tube to a pressure transducer (PMSET 2DT-XO 2TBG; Becton Dickinson, Singapore); the entire system was fluid-filled and infused at a rate of 1.5 mL/hour to maintain patency. Catheters were then taped to the skin to minimize movement during exercise. A probe for near-infrared spectroscopy was placed in proximity of the IMP catheter tip (NIRS, described below). Note that on occasion a patient’s symptoms were bilateral and in these cases IMP catheters were inserted in both legs. However, for experimental objectives NIRS was only measured on the one leg that was the most symptomatic and the IMP value for this limb only was reported in the present study.

### NIRS technique and cuff occlusion manoeuvres

A probe for near-infrared spectroscopy was secured to the skin above the TA in the direction of the muscle fibers. An adhesive shield with a window was attached to the skin to secure the probe. This placement site was in close proximity to the location of the IMP catheter tip. This site was confirmed by observing spikes in the IMP signal recording during soft palpations of the skin over the catheter tip. The placement of the shield was positioned by the same investigator on all occasions. Non-invasive measurements of local tissue oxygenation, deoxyhemoglobin (HHb), and oxyhemoglobin (HbO_2_) were made using near infrared spectroscopy (NIRS) (INSPECTRA Tissue Spectrometer-model 325, Hutchinson Technology Inc., MN, USA) capable of emitting and detecting light intensities at wavelengths 680,720,760 and 800 nm [12]. Prior to placing the probe on the TA it was inserted in a light scattering calibrator for capturing reference lights of all wavelengths. All tissue measurements were related to these reference data, thereby converting light intensity measurements to optical absorbance. Optical absorbance values were further processed into a scaled second derivative absorbance spectrum, whereby a measure of the oxygen saturation of haemoglobin was obtained and expressed as percent saturation (StO_2_%). The software supplied with the Inspectra device provides absolute values of StO_2_%. HHb and HbO_2_ are also reported and are relatively expressed from pre-set baseline values as arbitrary units. NIRS sampling was performed at 0.35 Hz. A pneumatic cuff (wide-contoured 18 cm) placed around the thigh was inflated to 250 mmHg to obtain full arterial occlusion (AO) and was maintained for 1 minute during which resting oxygen consumption was recorded as indicated by the rate of increase in HHb. AO during the l minute was confirmed by observation of a stable graphical trace for total haemoglobin. The cuff was abruptly released and the recovery of oxygenation was estimated by the rate of increase in StO_2_%. Applying occlusion with NIRS in this way to obtain estimates of oxygen consumption rate and recovery has been previously validated [6].

### Data processing

While the patient was supine, IMP at rest was recorded as the mean of 1 minute and resting StO_2_% (Rest StO_2_%) as the mean of 30 seconds just prior to cuff inflation. During cuff inflation (1-minute duration) the slope of increase in HHb (HHbslope_AO_) was calculated during the first 45 seconds and was representative of resting oxygen consumption. During AO, StO_2_% showed a trending decrease over time; with cuff release the slope of increase in StO_2_% (StO_2_%slope_AO_) was calculated during the first 25 seconds to represent oxygen delivery and the time it took for StO_2_% to reach one-half of the peak hyperaemic value (½RT-StO_2AO_) was calculated to represent recovery. For treadmill running, the absolute value of StO_2_% (Ex-StO_2_%) was calculated as the mean value of the last one minute just prior to stopping running, and the relative StO_2_% (ΔEx-StO_2_%) as the difference between resting StO_2_% and Ex-StO_2_%. To gauge recovery post exercise, the slope of increase in StO_2_% (i.e. Ex-StO_2_%slope) was calculated during the first 45 seconds after running to represent oxygen delivery and the time it took for StO_2_% to reach one-half of the peak hyperaemic value (½RT-StO_2ex_) was calculated to represent recovery. Of note the subjects were escorted back to the cot immediately after treadmill running (taking about 20 seconds), but the recovery data time references started immediately when the treadmill stopped. In addition, the absolute StO_2_% value at 90 seconds post exercise (Rec_90sec_) was expressed as a percent of resting StO_2_%.

### Statistical analysis

Statistical analyses were performed in SPSS 20.0 (IBM Inc., Chicago, Il, USA). The non-parametric Mann-Whitney U test was used for each of the anthropometric and NIRS outcome variables to test for differences between compartment syndrome groups and their respective controls (i.e. CECS-diabetics vs. CON-diabetics; CECS vs. CON). We were interested in whether our compartment groups differed in IMP, so a 2-way ANOVA was used to test group x time differences in IMP between CECS-diabetics and CECS. The level of significance was set to P<0.05. Data are presented in the Tables as means ± SD.

## Results

Anthropometric and blood pressure data did not differ between CECS-diabetics and CON-diabetics or between CECS and CON (Table 1). Males and females were equally represented for all groups.

For IMP, the ANOVA showed no overall differences between CECS-diabetics and CECS (P = 0.53), however IMP changed significantly over time (P < 0.001) but there was no group x time interaction (P = 0.99) (Table 2).

**Table 2:** IMP before and after treadmill running.

	CECS-D (n = 9)	CECS (n = 11)
IMP_REST_ (mmHg)	23 (8)	20 (4)
IMP_1-MIN_ (mmHg)	50 (21)	46 (17)
IMP_5-MIN_ (mmHg)	35 (14)	33 (9)

Data represent mean values (SD). REST–before running; 1-min– 1 minute after running; 5-min– 5 minutes after running. CECS-D–CECS-diabetics.

At rest (Rest StO_2_%) and during (HHBslope_AO_) and after (StO_2_%slope_AO_ and ½RT-StO_2AO_) arterial cuff occlusion there were no differences in NIRS variables between CECS-diabetics and CON-diabetics groups (P > 0.05 for all comparisons) (Table 3).

**Table 3:** NIRS variables at rest, for cuff occlusion, and during and after treadmill running.

	CECS-D (n = 9)	CON-D (n = 10)	CECS (n = 11)	CON (n = 10)
Rest StO_2_%	78.2 (12.6)	69.1 (20.8)	69.3 (16.2)	75.9 (11.2)
HHbslope_AO_^a^	0.3 (0.2)	0.3 (0.2)	0.2 (0.1)	0.3 (0.1)
StO_2_%slope_AO_^b^	3.1 (1.6)	3.4 (1.5)	1.8 (0.8)	3.8 (1.7) *
½RT-StO_2AO_ (seconds)	12.6 (10.6)	7.9 (3.5)	17.0 (11.5)	5.1 (3.1) *
Ex-StO_2_%	6.3 (8.6)	40.4 (22.0) *	11.3 (16.8)	34.1 (21.2) *
ΔEx-StO_2_%	-71.9 (19.1)	-28.7 (14.7) *	-58.1 (21.1)	-41.8 (20.1)
Ex-StO_2_%slope^b^	3.5 (2.6)	1.4 (0.8) *	3.1 (1.4)	1.9 (1.3)
½RT-StO_2ex_ (seconds)	31.8 (23.5)	40.8 (20.3)	14.7 (9.4)	20.7 (10.2)
Rec_90sec_ (%)	100.1 (19.4)	99.9 (10.9)	92.1 (11.3)	94.4 (10.8)

Data represent mean values (SD).

* P < 0.05 significance between compartment syndrome groups (CECS-D and CECS) and respective control groups (CON-D and CON). Units for slope calculations: ^a^au∙3.5∙sec^-1^ and ^b^StO_2_ x 3.5·sec^-1^. See text for detailed information of how variables were determined. CECS-D–CECS-diabetics; CON-D–CON-diabetics.

NIRS variables physiological interpretations:

Rest StO_2_%–oxygen saturation at rest prior to cuff occlusion

HHbslopeAO−resting muscle oxygen consumption rate

StO_2_%slopeAO−post cuff occlusion oxygen recovery rate

½RT-StO2AO−the time to half of the peak hyperemic oxygenation recovery after cuff release

Ex-StO_2_%–absolute deoxygenation during treadmill running

ΔEx-StO_2_%–relative deoxygenation during treadmill running

Ex- StO_2_%slope–post exercise (treadmill running) oxygen recovery rate

½RT-StO2ex−the time to half of the peak hyperemic oxygenation recovery after exercise

Rec_90sec_−magnitude of oxygen saturation recovery (relative to Rest StO_2_%) at 90 sec post exercise

**Fig 1:**
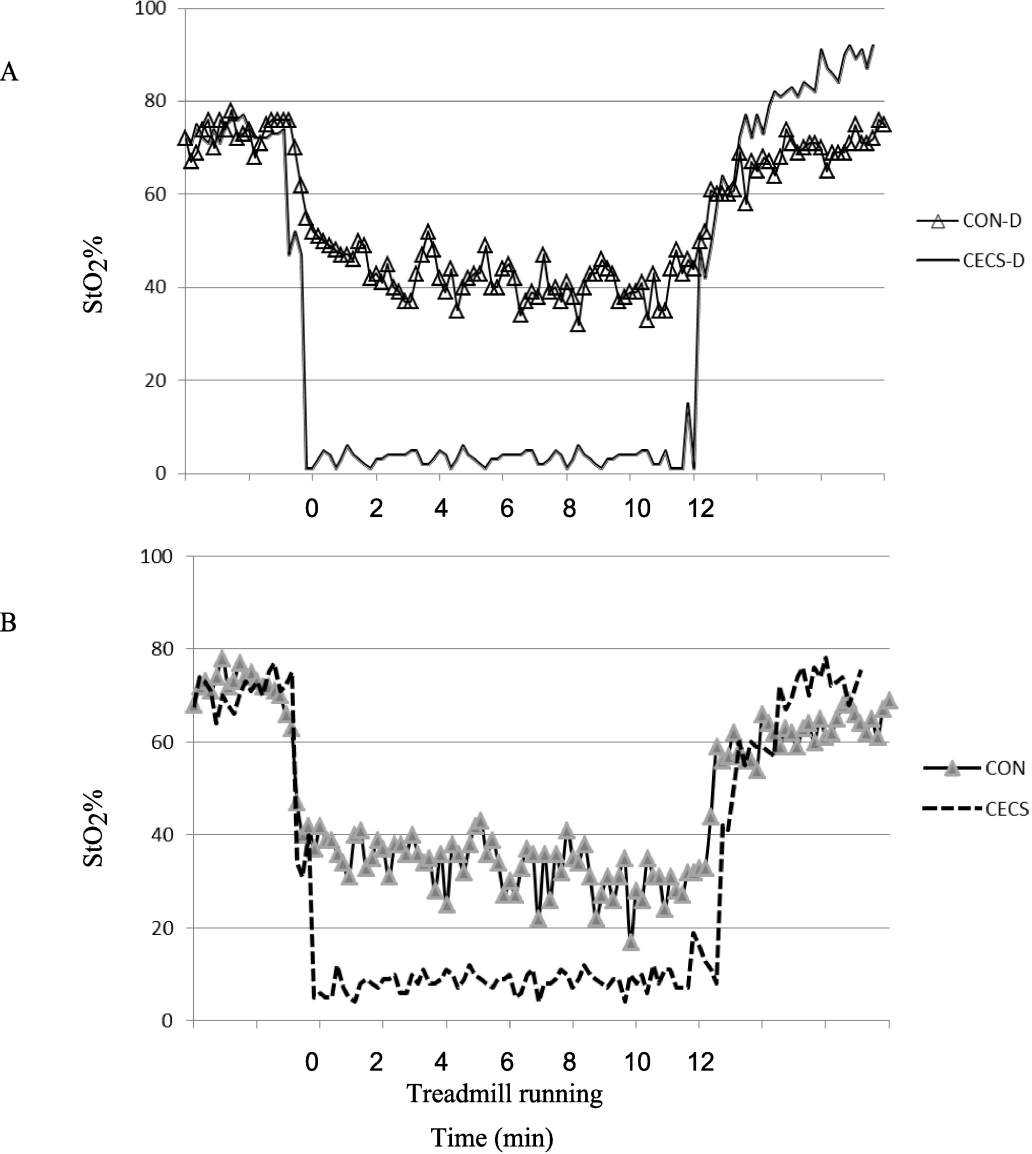
Typical NIRS curves before, during and after treadmill running for (A) CECS-D: diabetic patient with chronic exertional compartment syndrome and CON-D: diabetic patient without chronic exertional compartment syndrome; (B) CECS: non-diabetic patient with chronic exertional compartment syndrome and CON: healthy control subject. 0 min = start of running. CECS-D–CECS-diabetics; CON-D–CON-diabetics.

However, during exercise both the absolute (Ex-StO_2_%) and relative StO_2_% (ΔEx-StO_2_%) were significantly lower for CECS-diabetics (Table 3) (P < 0. 001 for both); this indicated more deoxygenation during exercise for CECS-diabetics (Fig 1A). Furthermore, the oxygen recovery rate post-exercise (Ex-StO_2_%slope) was significantly faster for CECS-diabetics (P = 0.04), but there was no difference between groups in the recovery time to half of the hyperaemic value (½RT-StO_2ex_) (P = 0.32). There was also no group difference in the percent recovery at 90 seconds post-exercise (Rec_90sec_) (P = 1.00).

For comparisons between CECS and CON there were no group differences in NIRS variables at rest (Rest StO_2_%) or during arterial cuff occlusion (HHBslope_AO_) (P > 0.05 for both) (Table 3). However, following arterial cuff occlusion both NIRS variables were significantly different between groups–StO_2_%slope_AO_ was at a slower rate for CECS (P = 002) and ½RT-StO_2AO_ was at a longer time for CECS (P = 0.002 for both). During exercise the absolute StO_2_% value (Ex-StO_2_%) was significantly lower for CECS (P = 0.02); however, there was no group difference in the relative StO_2_% (ΔEx-StO_2_%) (P = 0.13) (Fig 1B). The oxygen recovery rate post-exercise (Ex-StO_2_%slope) was not different between groups (P = 0.05, although this level was at a tendency for significance), and there was no difference between groups in the recovery time to half of the hyperaemic value (½RT-StO_2ex_) (P = 0.13). Furthermore, there was no group difference in the percent recovery at 90 seconds post-exercise (Rec_90sec_) (P = 0.92).

## Discussion

The present investigation is the first to apply near infrared spectroscopy (NIRS) to test for differences in oxygen kinetics between Type 1 diabetes patients with chronic compartment syndrome (CECS-diabetics) and diabetic patients without compartment syndrome (CON-diabetics). An important finding in the study was that during the treadmill exercise to reproduce pain symptoms, the CECS-diabetics group showed greater deoxygenation than that for the CON-diabetics group. This is in line with some previous studies that compared non-diabetic CECS patients with healthy controls, and also for these same group comparisons in the present study. Therefore, our hypothesis of greater deoxygenation for both compartment syndrome groups was confirmed. Based on the outcome of previous studies, in a recent review article by Boezeman et al. [7] the conclusion was reached that NIRS is a useful non-invasive method for assessing CECS. Therefore, our findings suggest that NIRS may be potentially useful for assessing CECS for diabetic patients.

The significantly greater deoxygenation during treadmill running for the CECS-diabetics group compared to the CON-diabetics group follows the trend previously reported for non-diabetics [3,13,14] as well as that seen in the present study for CECS versus CON. NIRS signals represent the dynamic balance between oxygen delivery and consumption. Therefore, group differences in StO_2_% during running could be due to higher oxygen consumption and/or impaired oxygen delivery for the CECS-diabetics and CECS groups compared to their respective control counterparts. The high IMP values at rest (in reference to reported values for healthy adult subjects of ≤ 10 mmHg [15]) for our CECS-diabetics (23 mmHg) and CECS (20 mmHg) groups were likely further increased to a magnitude during running that contributed to impaired oxygen delivery, but we cannot with certainty rule out whether there was higher oxygen consumption for the CECS-diabetics and CECS groups. However, we do know that at rest there was no difference in the oxygen consumption rate between the compartment syndrome groups and their respective control groups.

After exercise, the oxygenation recovery rate for the CECS-diabetics group was faster than that for the CON-diabetics group, and there was a tendency for significance for CECS versus CON. The ½ recovery time, however, was not different between either of the group comparisons, although on average the ½ recovery time was smaller (faster) for the CECS-diabetics and CECS groups. A presumed conjecture is that the ½ recovery time and oxygenation recovery rate are interrelated, but they may express separate physiological entities. While ½ recovery time is anchored to the post-exercise hyperaemic response, our slope calculations reflect the initial recovery at which primarily vascular components are restored (thus, is not influenced by the hyperaemic response). A previous study [16] attributed the post exercise oxygen recovery rate to the magnitude of deoxygenation during exercise, which would thereby explain the faster recovery rate for CECS-diabetics (and the tendency for CECS) compared to controls. The authors in the previous study discussed the production of hydrogen ions producing acidosis during the contraction to stimulate vasodilation. Taken at face value, our recovery data appear to be in contrast to Mohler et al. [3] and Zhang et al. [17] who both reported slower recovery after exercise in their compartment syndrome patients and van den Brand et al. [14] who found no group differences. Different manners in how recovery is calculated might contribute somewhat to the discrepancies between previous studies and our study.

The difference we found between CECS and CON in recovery variables following arterial occlusion (i.e. StO_*2*_%slope_*AO*_ and ½RT-StO_*2AO*_) is worthy of comment. This was not the case, however, for our CECS-diabetics versus CON-diabetics comparisons. To our knowledge, data of these types have not been assessed previously in NIRS compartment syndrome studies. While the drop in saturation during total cuff occlusion proximal to the site of NIRS measurement is used to assess oxygen consumption rate, the rate of increase post ischemia is indicative of the muscle microvascular function. In this line it could be surmised that for our CECS patients the muscle microvascularity is hampered compared to CON. We have no ready explanation for why there were no post cuff occlusion recovery differences between the diabetic groups, and we also feel it somewhat perplexing that our CECS-diabetics StO_*2*_slope_*AO*_ data were of the same magnitude that we found for our normal healthy subjects (i.e. CON). We recommend further investigation to assess the validity of our post occlusion recovery data for CECS versus CON.

In a previous study from our laboratory Edmundsson et al. [9] reported that intramuscular pressure (IMP) before and after treadmill running was significantly higher for CECS-diabetics patients compared to non-diabetic CECS patients. However, in the present study we found no group difference in IMP at any time point between these same types of patient groups. It is important to point out that in the previous study the clinical criteria for diagnosis of CECS was according to Pedowitz et al. [2], which was also applied in the present study since this is the routine procedure in our clinic. Another point is that in the Edmundsson et al. [9] the majority of patients were females, whereas females and males were equally represented in our study populations. Therefore differences in patient groups between the previous study and ours may contribute to the discrepancy between studies.

A consideration in all NIRS studies is the influence of adipose tissue thickness (ATT). During exercise if the ATT is too thick the NIRS signal will be blunted [18] indicating that the light of the probe does not penetrate into the muscle. The average depth of penetration of the 25-mm probe we used is considered to be 12.5 mm [19]. ATT was not measured in the present study but was reported over the tibialis anterior muscle to be 3–6 mm thick [20]. This means that the light penetration depth was likely sufficient to be within the muscle. Additionally, we affirm that none of our patients or subjects showed abnormal ATT thickness when we palpated for IMP catheter placement.

In conclusion, the Type 1 diabetic patients with compartment syndrome in the present study showed greater deoxygenation during treadmill running than Type 1 diabetic patients without compartment syndrome. This finding is in agreement with previous studies that compared non-diabetic CECS patients with healthy controls and for these same group comparisons in the present study. The fact that oxygenation differences were detected, suggests that NIRS could potentially be used as a diagnostic tool for assessing Type 1 diabetic patients suspected of CECS. However, to ascertain this, another type of study design would be needed; for example, a study that would entail correlating deoxygenation magnitudes with IMP magnitudes for a larger patient group than that used in the present study.
